# Autophagy and Ubiquitination in *Salmonella* Infection and the Related Inflammatory Responses

**DOI:** 10.3389/fcimb.2018.00078

**Published:** 2018-03-14

**Authors:** Lidan Wang, Jing Yan, Hua Niu, Rui Huang, Shuyan Wu

**Affiliations:** Department of Microbiology, Medical College of Soochow University, Suzhou, China

**Keywords:** *Salmonella*, autophagy, ubiquitination, effector protein, inflammation

## Abstract

*Salmonellae* are facultative intracellular pathogens that cause globally distributed diseases with massive morbidity and mortality in humans and animals. In the past decades, numerous studies were focused on host defenses against *Salmonella* infection. Autophagy has been demonstrated to be an important defense mechanism to clear intracellular pathogenic organisms, as well as a regulator of immune responses. Ubiquitin modification also has multiple effects on the host immune system against bacterial infection. It has been indicated that ubiquitination plays critical roles in recognition and clearance of some invading bacteria by autophagy. Additionally, the ubiquitination of autophagy proteins in autophagy flux and inflammation-related substance determines the outcomes of infection. However, many intracellular pathogens manipulate the ubiquitination system to counteract the host immunity. *Salmonellae* interfere with host responses via the delivery of ~30 effector proteins into cytosol to promote their survival and proliferation. Among them, some could link the ubiquitin-proteasome system with autophagy during infection and affect the host inflammatory responses. In this review, novel findings on the issue of ubiquitination and autophagy connection as the mechanisms of host defenses against *Salmonella* infection and the subverted processes are introduced.

## Introduction

According to the figures released by the Centers for Disease Control and Prevention, one million foodborne illnesses have been caused by *Salmonellae* in the United States annually, with 19,000 hospitalizations and 380 deaths (https://www.cdc.gov/salmonella/). *Salmonellae* most often cause gastroenteritis and typhoid fever ranging from mild to severe systemic infections that might be potentially life threatening. *Salmonella enterica* serovar Typhimurium (*S*. Typhimurium) and Typhi are the respective pathogens in *Salmonella* family that cause this debilitating condition. Additionally, increasingly reports demonstrated that *Salmonella* infection is related to many other diseases such as Gallbladder cancer (GBC), thus raising a worldwide major public health concern (Scanu et al., 2015). *Salmonella enterica* are transmitted by the fecal-oral route through contaminated food or water. The bacteria in the small intestine adhere to the mucosa and then preferentially invade the epithelial cells of the terminal ileum. Pathogens encounter the phagocytes including dendritic cells and macrophages after traversing the epithelial layer. They are ingested and can survive in the SCVs (*Salmonella*-containing vacuoles) of phagocytes. Moreover, the invading pathogens could spread to the mesenteric lymph nodes, and some disseminate to the reticuloendothelial cells of the extra-intestinal organs such as liver and spleen causing systemic infection (Keestra-Gounder et al., 2015).

Autophagy is a preserved process in eukaryotic cells that delivers cytoplasmic contents to the lysosome for degradation. The cytoplasmic materials such as damaged organelles, misfolded proteins, or intracellular microbes were engulfed by an isolation membrane (phagophore), which elongates to form a double-membraned vacuole (autophagosome), followed by the fusion with lysosome to form an autolysosome, in which the enclosed materials degraded to maintain cellular homeostasis (Deretic, 2011). Autophagy can be classified into selective and non-selective autophagy according to the degraded substance, and the selective clearance of pathogens by autophagy is regarded as xenophagy (hereafter referred as autophagy). Additionally, autophagy is a dynamic process with numerous ATG (autophagy related) proteins and autophagy adaptors involved, the host autophagy flux could be influenced by the interaction between pathogens and them. Once infected with *Salmonella*, autophagy can be induced rapidly which plays a pivotal role in the elimination of bacteria and the process of autophagy can even affect the following innate and adaptive immune responses to pathogens (Gomes and Dikic, 2014). Chaperone-mediated autophagy (CMA) is involved in transportation of specific cytosolic proteins to lysosomes for degradation. Interesting, CMA does not participate in the clearance of *Salmonellae*, on the contrary, nutrients in favor of intracellular *Salmonellae* growth can be supplied by CMA-dependent pathway (Singh et al., 2017).

Ubiquitination is an enzymatic cascade reaction by which ubiquitin (Ub) is covalently bound to protein substrates, mediated by E1 (Ub-activating enzyme), E2 (Ub-conjugating enzyme), and E3 (Ub ligase enzyme), and this process can be reversed by deubiquitinases (DUBs). Ubiquitination is one of the pivotal “eat-me” signals, initiating the process of autophagy. Ub contains eight distinct chains, seven are lysine residues including K6, 11, 27, 29, 33, 48, and 63. The carboxy-terminal glycine of Ub attaches to an active-site cysteine of E1 through a reactive thioester bond. The activated Ub is transferred to the E2 by an analogous reaction, and the E3 catalyzes the attachment of the Ub to a lysine in the target protein. The existence of mono-Ub and poly-Ub was determined by the relative proportion between the E3 and the target protein. Besides, a special linear M1 (methionine)-ubiquitination chain was identified as the eighth Ub chain, which was generated by the formation of a peptide bond between the amino-terminal methionine residue of the preceding Ub molecule and the carboxy-terminal glycine (Walczak et al., 2012). These distinct Ub chains have different effects on the function of protein substrates. In terms of autophagy, different Ub chains show different affinities for autophagy receptors. For example, ubiquitination of the autophagy receptor p62 (the Ub sensor SQSTM1), was suggested to display a preference for K63 chains over K48 (Gomes and Dikic, 2014). The Ub ligase Smurf1 plays a role in autophagy of intracellular bacteria such as *Mycobacterium tuberculosis* (*M. tuberculosis*, Mtb) and *Listeria monocytogenes* (*L. monocytogenes*). Smurf1 recruits the proteasome, K48 Ub chain, and the autophagy machinery components to Mtb therefore restricts the replication of bacteria in macrophages both *in vitro* and *in vivo*. It was noticeable that the function of Smurf1 in autophagy requires K48-linked ubiquitination rather than K63 (Franco et al., 2017). The ubiquitination pathway and involvement of different enzymes in ubiquitination cascades are capable of regulating the metabolism and function of proteins as well as the inflammation, immunity and so forth. Eldridge et al. uncovered that the UBE2L3 is a E2 Ub conjugating enzyme which plays an essential role in inflammation. It can be targeted by inflammasomes and leads to the activation of caspase-1 as well as the production of mature IL-1β (Eldridge et al., 2017). DUBs that are specific toward topologies of different Ub chains can remove Ub moieties and shape the proteins fate. Small molecule inhibitors with DUB activity such as USP5 and UCH-L5 were identified to regulate the assembly and activation of inflammasome (Lopez-Castejon et al., 2013; Kummari et al., 2015).

In addition, ubiquitination is one of the post translational modifications (PTMs), which modifies virulence factors such as Type III secretion system (T3SS) effector proteins to manipulate its interplay with host cells. Reciprocally, some effector proteins act as Ub ligase enzyme or DUBs affecting autophagy flux or inflammatory responses. Therefore, targeting ubiquitination of proteins and ubiquitination pathways might be helpful for the resistance against *Salmonella* infections through modulating the process of autophagy and inflammation (Salomon and Orth, 2013). As the host defenses, autophagy and inflammation both play an essential role in resisting pathogens and they have some close ties with each other. On one hand, mediators of inflammation including innate immune receptors, inflammatory cytokines and inflammation-related transcription factors can regulate autophagy. On the other hand, autophagy can regulate inflammatory responses by modulating the activation of inflammasomes, the polarization of immune cells and the secretion of inflammatory cytokines.

To counteract the host immunity, *Salmonella* can subvert the Ub and autophagy pathways by delivery of several bacterial effectors into the cytosol to evade the host defenses. In this review, we discuss mechanisms by which Ub and autophagy work on the resistance against *Salmonella* infections as well as the related inflammatory responses. Meanwhile, we address how *Salmonellae* escape from either autophagy or inflammation by disturbing the process of ubiquitination.

## Ubiquitin-dependent autophagy in the elimination of *Salmonella*

**Figure 1:**
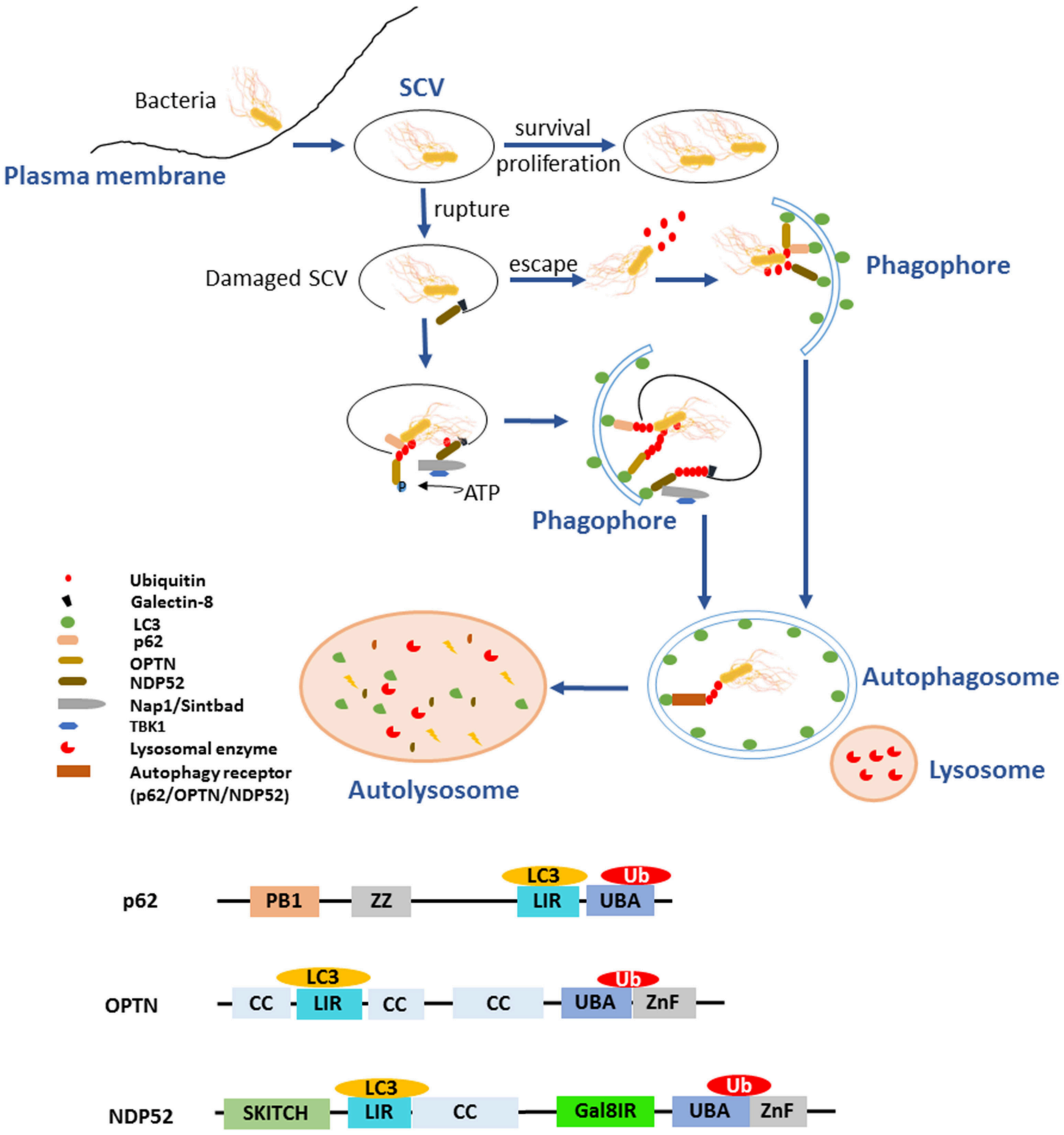
Ubiquitination and autophagy during *Salmonella* infection. After invading into host cells, *Salmonellae* reside in *Salmonella*-containing vacuoles (SCVs), the modified phagosomal compartment. There are different endings for the bacteria depending on the integrity of SCVs. The SCVs are the protective compartment for *Salmonellae* survival and proliferation, while they could be damaged by needle-like apparatus T3SS-1. With the SCVs ruptured, a portion of *Salmonellae* escaped and exposed to the cytoplasm. Cytosolic bacteria are ubiquitinated rapidly as a “eat-me” signal leading to the recruitment of autophagy receptors such as p62/SQSTM1, OPTN, and NDP52 to initiate the process of autophagy and being degraded. The remaining bacteria in the damaged SCVs can also be eliminated by autophagy with another “eat-me” signal, the exposed galectin-8. When the integrity of SCVs is lost, exposure of galectin-8 can recruit autophagy receptor NDP52. NDP52 binds the adaptor proteins Nap1 and Sintbad and recruits TBK1 nearby the cytosol-exposed bacteria, TBK1 phosphorylates OPTN thereby leading to enhanced affinity for LC3 and initiates autophagy. All the autophagy receptors p62, OPTN, and NDP52 contain LC3-interacting region (LIR) and ubiquitin-associated (UBA) domain, which bridge Ub-coated bacteria to the autophagy modifiers LC3. In addition, the receptors with special domain enrich their function on autophagy process. ZZ domain of p62 can promote the delivery of itself and cargos to the autophagosomes. Gal8IR region of NDP52 is involved in the recognition of autophagy.

Autophagy plays an essential role in host responses to various environmental stimulus as a housekeeping, especially the invasion of bacteria. The clearance of *Salmonellae* by autophagy is a kind of selective autophagy that is involved in the process of recognition, recruitment, and elimination (Gomes and Dikic, 2014). Ubiquitination plays a crucial role in bacterial recognition and targeting, and even modifies the core autophagy components, regulating the clearance of pathogens by autophagy flux (Grumati and Dikic, 2017). Upon exposure to cytoplasm, *Salmonellae* are ubiquitinated immediately and then shipped to the autophagy receptors as cargoes. LC3, the autophagy modifiers (microtubule associated protein 1 light chain 3, MAP1LC3, abbreviated as LC3 henceforth) is located on the autophagosomal membrane. LC3-interacting region (LIR) motif is a domain contained in the autophagy receptors p62, NDP52 (nuclear domain 10 protein52), and OPTN (Optineurin) (Gomes and Dikic, 2014). All the receptors also hold a similar domain that functions as the Ub-binding. The pathogens interact with LIR through Ub, inducing the formation of phagophore, then the subsequent formation of autophagosome and the degradation of bacteria in autolysosome (Figure 1). Accordingly, ubiquitination is indispensable in clearance of bacteria by autophagy. However, pathogens evolve numerous strategies to subvert autophagy to protect them from elimination.

As facultative intracellular bacteria, *Salmonellae* could reside in both phagocytes and non-phagocytic cells such as epithelial cells. T3SSs are used by bacteria to export proteins from bacterial cytosol into eukaryotic cells. These bacterial effector proteins involving in the invasion of the epithelial barrier and the pathogenesis of infection. The mechanism of T3SS delivery is a contact-dependent process and highly conserved, characterized by the formation of pores, or translocon on cytomembranes of host cells when bacteria contact with them and through which the effector proteins are delivered into the host cell (Song et al., 2017). *S*. Typhimurium possesses two distinct T3SSs, located on *Salmonella* Pathogenicity Islands 1 and 2 (SPI1 and SPI2), called T3SS-1 and T3SS-2, respectively (Burkinshaw and Strynadka, 2014). After invading the host cells, *Salmonellae* reside and replicate inside the modified phagosomes that termed SCVs. It seems to be helpless for the host to respond to the *Salmonellae* within SCVs. Nevertheless, the needle-like apparatus T3SS-1 can cause damage to the SCVs, which gives rise to multiple consequences for the pathogen. The majority of invading *S*. Typhimurium enter the SCVs for replication, while other portions can damage the SCVs by T3SS (Owen and Casanova, 2015). Subsequently, a portion of *S*. Typhimurium manages to escape from the SCVs and proliferates in the cytosol. These bacteria that either egress into cytosol or remain residing in damaged SCVs are rapidly ubiquitinated and then targeted by autophagy receptors (p62, OPTN, NDP52) to the autophagosome which ultimately fuses with the lysosome in which degradation of the bacteria occurred (Figure 1) (Birmingham and Brumell, 2006; Cemma et al., 2011). SifA is a T3SS-2 effector protein with N and C-terminal domain supporting *Salmonella* virulence. Its N-terminal domain is critical for maintaining the integrity of SCVs by inducing tabulation of the SCV and binding the mammalian kinesin-binding protein SKIP, which is critical for bacterial proliferation and evading inflammation mediated by caspase-11. Its C-terminal domain possesses the activity of guanine nucleotide exchange factor contributing to SifA virulence independent of its N-terminal domain (Beuzón et al., 2000; Ohlson et al., 2008; Aachoui et al., 2013a; Zhao et al., 2015). SifA seems essential against the initiation of autophagy to some extent due to its role in maintaining the integrity of SCVs. As a modulator of phosphoinositide 3-phosphate (PI(3)P) levels on early and recycling endosomes by dephosphorylation, the phosphoinositide 3-phosphatase myotubularin 4 (MTMR4) plays a role in survival of *S*. Typhimurium. Additionally, the regulation of PI(3)P is also requisite for the stability and integrity of SCVs, which may modulate the process of autophagy (Teo et al., 2016).

Undoubtedly, there are more than one possible autophagy-dependent pathway of pathogen degradation. Either Ub-dependent or -independent mechanism is involved in pathogen targeting. In particular, Ub, a well-known signal for the degradation of polypeptides in the proteasome, together with autophagy are of great importance for bacterial degradation. Certainly, both the Ub-proteasome system and autophagy play crucial roles in *Salmonella* infection. It has been found that a portion of intracellular *S*. Typhimurium exposed to the cytoplasm is rapidly ubiquitinated, leading to the recruitment of several autophagy receptors, including NDP52 and p62, as well as the TANK-binding kinase 1 (TBK1) (Rogov et al., 2013). Analysis using immunofluorescence microscopy shown that cytosolic *Salmonellae* are recognized by p62, NDP52, OPTN, Nap1 (NF-κB activating kinase-associated protein 1), Sintbad (also known as TBKBP), and TBK1 (TANK binding kinase1) (Ivanov and Roy, 2009). p62 was identified as the first mammalian autophagy adaptor, and it participates in many autophagy processes, including the selective degradation of cytosolic proteins and the clearance of intracellular pathogens. p62 has two domains, the Ub-associated (UBA) domain of p62 binds Ub-coated *Salmonellae* and LIR motif of p62 binds LC3, through which p62 facilitates the recognition of *Salmonellae* by autophagy (Fujita and Yoshimori, 2011). p62 also involves in a novel pathway regulating autophagy by inducing autophagosome biogenesis. p62 can promote the delivery of itself and cargoes to the autophagosome through its ZZ binding domain (Figure 1). In addition to the regulating of autophagy process, p62 may also mediates the crosstalk between autophagy and the Ub-proteasome system (Cha-Molstad et al., 2017). NDP52 is another receptor that recognizes ubiquitinated *Salmonellae* and is associated with bacterial clearance via the autophagy pathway (Ivanov and Roy, 2009). NDP52 binds the adaptor proteins Nap1 and Sintbad and recruits TBK1 nearby the cytosol-exposed bacteria (Ishimura et al., 2014). Thurston et al. observed that the host proteins Nap1 and Sintbad colocalize with ubiquitinated *Salmonellae*, which may restrict the growth of *Salmonellae* in eukaryotic cells. NDP52 also binds LC3, and the silencing of NDP52 impairs the clearance of *Salmonellae* by autophagy (Figure 1) (Thurston et al., 2009). Galectin-8, a cytosolic lectin, is a bacterial restriction factor that can induce NDP52-mediated autophagy. Upon the damage of SCVs, the following exposed host glycans recruit and bind abundant galectin 8, which could be recognized by NDP52 and then the NDP52-galectin-8 complex targets the Ub-associated *S*. Typhimurium to initiate autophagy (Figure 1) (Thurston et al., 2012). TANK-binding kinase 1 (TBK1) is a member of the inhibitor of nuclear factor κB kinase (IKK) family and participates in innate immune responses. The activity of TBK1 is required to induce the innate immune responses against viral infection through enhancing the expression of type I interferons and other antiviral proteins (Weidberg and Elazar, 2011). Radtke et al. reported that TBK1 restricts the replication of cytosolic *Salmonellae*, which is distinct from its role in virally infected cells (Radtke et al., 2007). On one hand, TBK1 confines the expression of the water channel aquaporin-1 that is crucial for the integrity of SCVs, which results in *Salmonellae* exposed to the cytosol followed by the accumulation of poly-ubiquitinated proteins on the surface of bacteria, and then facilitates the elimination of *Salmonellae* by autophagy (Ivanov and Roy, 2009). On the other hand, TBK1 phosphorylates OPTN at Ser177, thereby leading to enhanced affinity for LC3. In addition, it demonstrates that TBK1 can also phosphorylates S473 and S513 in OPTN to facilitate Ub chain binding (Heo et al., 2015). It has been revealed that TBK1 is indispensable for the recruitment of WIPI2, a PI(3)P-binding component of upstream autophagy (Thurston et al., 2016). OPTN also has two domains, UBA and LIR motif (Figure 1). OPTN can bind Ub-coated cytosolic *Salmonellae* and the depletion of OPTN in HeLa cells promotes bacterial replication, suggesting that OPTN mediates clearance of pathogens by autophagy. Beyond its function as an autophagy receptor, OPTN is closely related to maturation of autophagosomes to autolysosomes, indicating that it promotes the autophagy in antimicrobial immunity (Thurston et al., 2016). Weidberg et al. verified that NDP52 and OPTN function together to mediate *Salmonellae* degradation. It is possible that NDP52 functions upstream of OPTN and locally activates TBK1 to enable recruitment of LC3 by OPTN (Weidberg and Elazar, 2011). NDP52 plays a dual function during autophagy. It can target bacteria to nascent autophagosomes at the initiation of autophagy and ensure subsequent pathogen degradation by regulating maturation of autophagosome, respectively (Verlhac et al., 2015). Intriguing, as has been mentioned above, both OPTN and NDP52 are able to facilitate autophagosome maturation, which depend on myosin VI adaptor proteins, accelerating the autophagy-dependent clearance of *S*. Typhimurium (Tumbarello et al., 2015).

Considering that the autophagy process is associated with cytoskeletal machinery and integrity of the membrane structure, the dynein clusters may also play essential roles in antimicrobial autophagy flux (Sotthibundhu et al., 2016). SifA-SKIP interaction modulates the activity of the molecular motor kinesin, which may be the fundamental to the formation of autophagosomes. Contradictory to the viewpoint mentioned above that SifA is helpful for evading activation of autophagy, it may have facilitation toward autophagy flux. Furthermore, a latest study shows that Dynein motors physically cluster into lipid rafts on the membrane of a phagosome along with which matures inside the cell (Rai et al., 2016). The geometric organization in clusters makes many motors united together to drive transportation of the phagosome, and promote phagolysosome fusion. Therefore, the activity of the molecular motor kinesin is also critical for elimination of *Salmonellae* by accelerating the formation of autophagosomes.

It was demonstrated that E3 Ub ligases control substrate selectivity in the ubiquitination cascades. Recently, RNF166 are identified as E3 Ub ligases that are required for adaptor protein recruitment and LC3-bacteria colocalization. RNF166 has a dual role in controlling the recruitment of Ub as well as p62 and NDP52 to bacteria. Though there are no valid explanations for this phenomenon, it was speculated that different E3 ligases promote different Ub chain linkages (Heath et al., 2016). The HECT E3 Ub ligase NEDD4 is identified as an LC3-interactive protein, regulating autophagy through a conserved LIR domain (Sun et al., 2017). As adaptor protein, p62 is one of the core autophagy components and the modulation of p62 by ubiquitination may determine the process of autophagy. The ubiquitination of p62 can disrupt dimerization of its UBA domain and then its ability to recognize poly-ubiquitinated cargoes was accessible to selective autophagy. The interaction between Ub conjugating enzyme UBE2D2/3 and p62 is essential for the upregulation of Ub homeostasis (Ub^+^), which promotes recognition of poly-ubiquitinated cargoes and autophagy flux (Peng et al., 2017). The ubiquitination of UBA domain of p62 by the Keap1/Cul3 complex augments its activity of sequestering ubiquitinated cargoes and recruits them to the growing autophagosome (Lee et al., 2017). The influence of p62 ubiquitination on autophagy flux related to *Salmonella* infection has not been thoroughly studied previously. These Peng and Lee's remarkable findings shed new light on the predominant effect of ubiquitination on autophagy flux.

Several mechanisms of antimicrobial autophagy are described to impact directly or indirectly on *Salmonellae* clearance. Initiation of autophagy is determined by various autophagy adaptors (p62, NDP52, OPTN etc.) and the affinity of binding partners (galectin 8, TBK1, and LC3). Meanwhile, the maturation of autophagosome is implicated in this process. In order to illuminate the Ub-dependent autophagy in the elimination of *Salmonellae*, attention should be paid to the ubiquitination as well as the detailed process of autophagy.

## *Salmonella* T3SS effector proteins affect the antibacterial inflammation and autophagy by triggerring the ubiquitin pathway

**Figure 2:**
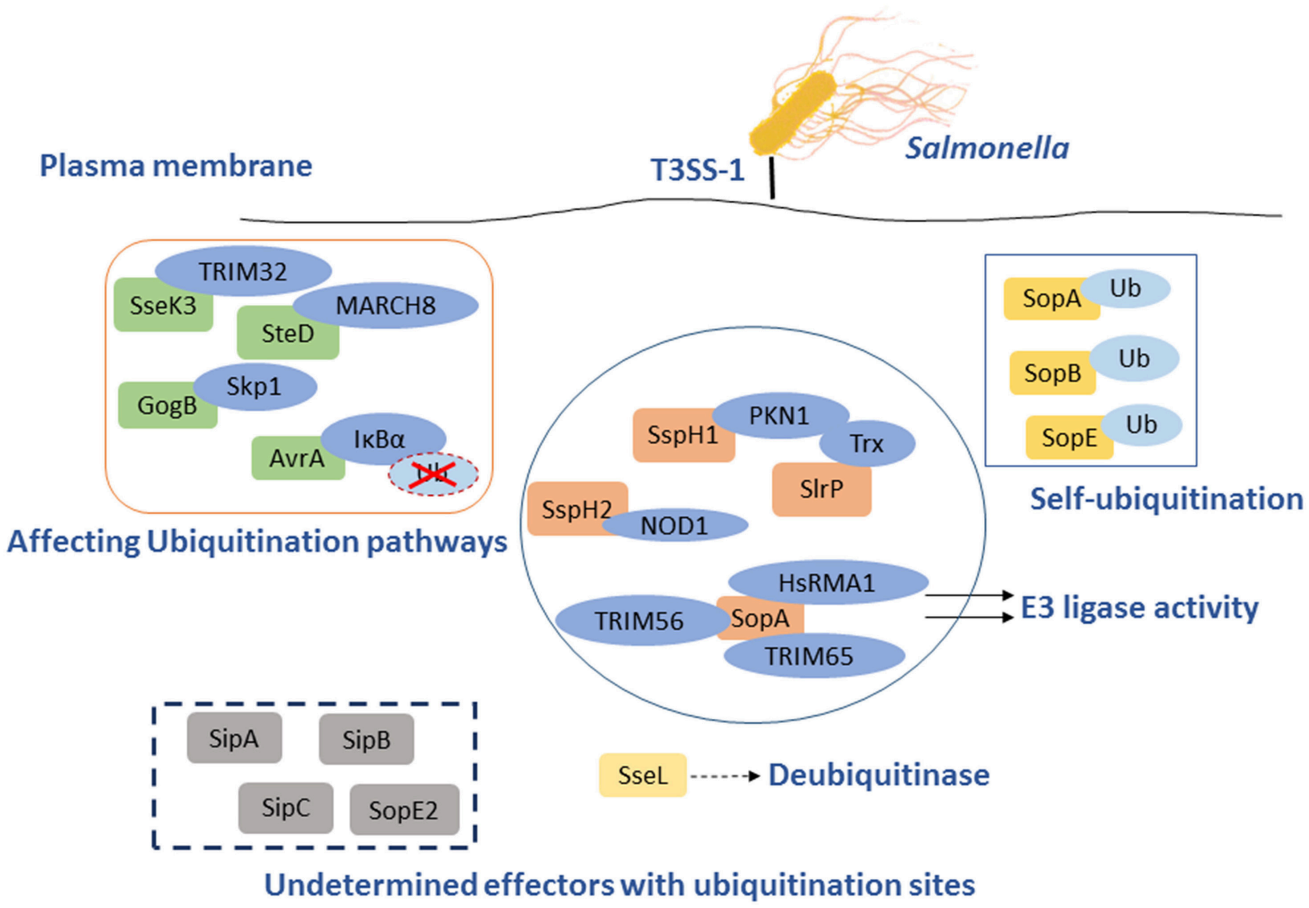
*Salmonella* T3SS effector proteins that interact with the eukaryotic ubiquitin system. Some effectors act as E3 ligase interacting with host proteins to inhibit NF-κB pathways, such as SopA, SlrP, SspH1, and SspH2. The others serve as DUB to play a role on the responses to infection through Ub pathway, SseL is the best example. GogB and AvrA are effectors that interact with the host Ub pathways following diversity mechanism to impact the immune system. Ssek and SteD can affect host immune responses by interacting with E3 Ub ligase TRIM32 and MARCH8, respectively. Furthermore, SopB, SopA, and SopE could also be self-ubiquitinated to influence the antimicrobial process. However, several effectors with ubiquitination sites remain to be determined.

*Salmonella* T3SSs secrete and deliver effector proteins that are closely related to pathogenesis, including invasion of the host cells, pyroptosis, intracellular survival, and evasion of host immune responses. Some effectors, such as SipA, SipC, SopB, SopD, SopE, and SopA, participate concertedly in the invasion process and bacterial internalization (Rossignol et al., 2014). Some other effectors contribute to the modulation of the inflammatory responses, most of which are relevant to ubiquitination pathway or Ub. Several SPI-1 and SPI-2 effectors interfere directly with Ub-related processes, such as E3 ligases SopA, SspH1, SspH2, and SlrP (Ria et al., 2009), and the DUB SseL (Figure 2) (Rytkönen et al., 2007). Furthermore, SPI-1 T3SS effectors SopA, SopE, and SopB/SigD are all translocated and then ubiquitinated, some of which can work together with Ub to interfere with the immune responses.

SopA mimics mammalian HECT E3 Ub ligase and preferentially utilizes the host UbcH5a, UbcH5c, and UbcH7 as E2 Ub conjugating enzymes, which are involved in stimulation of inflammation (Diao et al., 2008). It has been reported that SopA interacts with human RMA1 (HsRMA1), a RING finger family E3 Ub ligase, promoting the escape of *Salmonellae* from the SCVs. In an HsRMA1-dependent manner, *sopA* mutant escapes less frequently from the SCVs into cytosol than wild-type *Salmonella* does (Zhang et al., 2006). SopA can stimulate innate immune signaling by targeting Tripartite motif (TRIM) 56 and TRIM65, two host E3 Ub ligases (Kamanova et al., 2016). As an HECT-like E3 Ub ligase, SopA can also ubiquitinate TRIM56 and TRIM65, resulting in their proteasomal degradation during infection (Figure 2 and Table 1) (Fiskin et al., 2017). The *Salmonella* effectors SspH1 and SspH2 belong to the NEL family of proteins that mediate E3 Ub ligase activity (Table 1). These effectors mimic eukaryotic proteins to subvert the immune responses. SspH1 acts as an E3 Ub ligase, and interacts with the mammalian protein kinase PKN1 to inhibit the NF-κB signaling pathway (Keszei et al., 2014). It was shown that SspH2 could modulate innate immunity in both mammalian and plant cells using model systems in a cross-kingdom approach. In mammalian cell culture, SspH2 significantly enhanced Nod1-mediated IL-8 secretion when transiently expressed or bacterially delivered (Table 1) (Bhavsar et al., 2013). In a nucleotide-binding leucine-rich repeat receptor (NLR) model system, SspH2 causes phenotypic modifications, which require its catalytic E3 Ub ligase activity and interaction with the conserved host protein NLR co-chaperone SGT1 (Bhavsar et al., 2013). SlrP is another E3 Ub ligase that employs thioredoxin as a substrate. Thioredoxin plays an important role in redox regulated signaling events and participates in numerous physiological processes (Figure 2 and Table 1) (Cordero-Alba and Ramos-Morales, 2014). SlrP contains 10 copies of a leucine-rich repeat (LRR) signature and is translocated into host cells by both T3SS-1 and T3SS-2, which influences inflammation responses in the early and late stages of infection (Table 1). The IpaH family effectors were mainly characterized in *Shigella*, however, these proteins are widely conserved among animal and plant pathogens, including *Salmonella* effectors SspH1, SspH2, and SlrP. They are characterized as novel E3 ligase (NEL) enzymes, which recruit host substrates for ubiquitination through a LRR domain (Rohde et al., 2007; Keszei et al., 2014). *Shigella* effectors IpaH possess the activity of E3 ligase activity, target and inhibit NF-κB signaling by manipulating the host Ub system and leading with downregulation of host inflammatory responses. In the same way, we speculate that *Salmonella* T3SS effectors SspH1, SspH2, and SlrP may also interact with host Ub to regulate the inflammatory responses (Ashida et al., 2015).

**Table 1:** *Salmonella* T3SS effector proteins that interact with the eukaryotic ubiquitin system.

**Effector**	**Activity**	**Interacting partner**	**Consequence of the interaction**
SopA	HECT-like E3 ubiquitin ligase	Human RMA1 (HsRMA1)	Promote the escape of *Salmonella* from the SCVs
		E3 ubiquitin ligaseTRIM56 and TRIM65	Result in their proteasomal degradation
SspH1		Mammalian protein kinase PKN1	Inhibit the NF-κB pathway
SspH2	E3 ubiquitin ligase	NOD1	Enhance IL-8 secretion
SlrP		Thioredoxin	Regulate signaling events and physiological processes
AvrA	Ubiquitin-like acetyltransferase/cysteine protease	Remove Ub from Ub-IκBα	Interfere with the NF-κB pathway
GogB	Inhibit IκBα degradation	Skp1-Cullin1-F-box (SCF) E3 ubiquitin ligase	Inhibit IL1 production and interferes with NF-κB activation
SseK3	Unknown	E3 ubiquitin ligase TRIM32	Modulate NF-κB activation
SteD	Unknown	E3 ubiquitin ligase MARCH8	Inhibit dendritic cell-mediated activation of T cells
SseL	Deubiquitinase	Ubiquitin	Inhibit autophagy by reducing ubiquitinated aggregations
SopA			Degrade through the HsRMA1-mediated ubiquitination pathway
SopB	Self-ubiquitination		Promote neutrophil accumulation at the site of infection
SopE			Degrade through proteasome-mediated pathway
SipA			
SipB	Undetermined	Undetermined	Undetermined
SipC			
SopE2			

The virulence-associated effector protein AvrA of *Salmonella* is a Ub-like acetyltransferase/cysteine protease, which interferes with the first line of immune defense. AvrA interferes with the NF-κB pathway by removing Ub from Ub-IκBα or via the acetylation of specific mitogen-activated protein kinase kinases (MAPKKs). AvrA mitigates IL-8 production and thereby inhibits the inflammatory responses of the host against infectious agents (Figure 2 and Table 1) (Giacomodonato et al., 2014). GogB is a substrate of both T3SS-1 and T3SS-2 in some *Salmonella* strains. GogB has an N-terminal LRR domain similar to those of LRR-containing *Salmonella* effectors SspH1, SspH2, and SlrP (Quezada et al., 2009). GogB inhibits IL1 production and interferes with NF-κB activation by inhibiting IκBα degradation through its interaction with the Skp1-Cullin1-F-box (SCF) E3 Ub ligase, leading with a limited activation of innate immune defenses in the host (Figure 2). Anti-inflammatory properties of GogB are important for the bacteria to reach optimal infection density in host tissues and to limit the tissue damage associated with a prolonged active inflammatory responses. These results suggest that GogB may play an essential role in downregulating the host inflammatory responses during infections (Pilar et al., 2012). In addition, the T3SS effector SseK3 is relevant to *Salmonella* infection through a novel molecular interaction with an E3 Ub ligase, TRIM32 (Yang et al., 2015). Like other E3 Ub ligases, TRIM32 is able to regulate its own activity by auto-ubiquitination. The relevance of the SseK3-TRIM32 does play a critical role in *Salmonella* infections and the modulation of NF-κB activation via ubiquitination pathway, even though the specific mechanism is yet unclear (Figure 2). MHC class II molecules play a major role in adaptive immune responses by presenting antigens to the CD4 restricted T cells. *Salmonellae* are able to interfere with the expression of MHC in both professional and non-professional antigen presenting through ubiquitination of HLA-DR in the cell surface, thus depressing the MHC antigen presentation. And this is dependent on T3SS-2 (Lapaque et al., 2009). The T3SS-2 effector SteD was proved to be required and sufficient for suppressing the activation of T cell during *Salmonella* infections. This process is also closely related to the ubiquitination pathway. SteD binds surface-localized mature MHC class II (mMHCII) as well as the host E3 Ub ligase MARCH8 (membrane associated ring-CH-type finger 8). It employs MARCH8 to promote mMHCII ubiquitination and surface depletion, leading to inhibition of dendritic cell-mediated activation of T cells (Figure 2 and Table 1) (Bayer-Santos et al., 2016). In addition to their action in adaptive immune responses, endosomal MHC class II (MHCII) molecules were proved to regulate innate immunity via sharp tuning the TLR4 signaling pathway. However, MARCH1 E3 Ub ligase exerts an MHCII-independent effect that accommodating the innate immunity based on ubiquitination (Galbas et al., 2017). Although AvrA, GogB, SseK3, and SteD are the effectors without enzymatic activity of ubiquitination, they can affect the inflammatory responses by degrading Ub or interacting with different E3 Ub ligases.

Intravacuolar *S*. Typhimurium induces SPI-2 T3SS-dependent ubiquitination of protein aggregations during infection, the formation of ubiquitinated aggregations are subsequently subjected to autophagy (Szeto et al., 2006). However, this effect may be counteracted by the SPI-2 T3SS DUB SseL, which has a preference for K63-linked chains and contributes to macrophages death (Coombes et al., 2007; Rytkönen et al., 2007; Le Negrate et al., 2008). SseL DUB activity reduces the Ub level of p62 and LC3 in SCV-associated aggregates, suggesting that SseL reduces autophagy flux in infected cells. Previous studies demonstrated that SseL may regulate antigen presentation in infected macrophages and dendritic cells, contributing to the virulence of *S*. Typhimurium in mice and reducing the innate immune responses *in vivo*. Replication of *SseL* deletion-mutant *Salmonella* in macrophages is significantly decreased compared with that of wild-type bacteria. Overall, it could be concluded that SseL decreases autophagy flux and favors intracellular *Salmonellae* replication by interfering with ubiquitination pathway (Mesquita et al., 2012).

Beyond that some T3SS effectors have a vital influence on *Salmonella* infection by interacting with ubiquitination pathway mentioned above, the T3SS effectors SopA, SopE, and SopB are able to be self-ubiquitinated (Figure 2 and Table 1). SopA and SopE are degraded through the HsRMA1-mediated ubiquitination pathway and proteasome-mediated pathway, respectively (Zhang et al., 2005). Upon infection, SopB is ubiquitinated in the host cell and then required for neutrophil accumulation at the site of infection in the intestine. Ubiquitination of SopB is necessary for appropriate vesicle trafficking of the SCVs, and it activates RhoG to remodel actin, resulting in membrane ruffles. Self-ubiquitination of SopB leads to recruitment of Rab5 to the SCVs, which normally participates in the maturation of phagolysosomes (Patel et al., 2009). Ruan et al. showed that UbcH5c/TRAF6 is a Ub ligase involved in the ubiquitination of SopB (Ruan et al., 2014).

Many ubiquitination sites of different SPI-1 effectors were detected by quantitative ubiquitination site profiling using diGly proteomics, such as SipA, SipB, SipC, SopE2, SptP, SopA, GogB, SopB, and SopE (Fiskin et al., 2016). The role of the effectors SptP, SopA, GogB, SopB, and SopE have been clearly identified during *Salmonella* infections by the characterization of their ubiquitination sites and their interacting partners, while the role of the rest effector proteins such as SipA, SipB, SipC, and SopE2 remains to be determined (Figure 2 and Table 1). Ubiquitination is a process involves in various antimicrobial mechanisms such as autophagy and inflammation as mentioned above, the novel identified effectors with ubiquitination site may play potential roles in *Salmonella* infection.

## The inflammatory responses to *Salmonella* infection and its interaction with autophagy as well as ubiquitination

In response to *Salmonella* infection, inflammation of the host is stimulated by the production of the pro-inflammatory cytokines and the activation of inflammatory caspases. Caspase-1 and caspase-11 in mice, and caspase-1, caspase-4, and caspase-5 in humans are the inflammatory caspases, and they are activated through the stimulation of either the NLRC4 or NLRP3 inflammasome (Martinon and Tschopp, 2007). Apart from its direct antimicrobial mechanism through lysosomal degradation, the initiation of autophagy has a great impact on the host innate and adaptive immunity. In recent years, a number of studies demonstrated that autophagy can modulate the differentiation of immune cells including neutrophils, T& B lymphocytes via a metabolism pathway (Riffelmacher et al., 2017) (Bhattacharya et al., 2015). Engulfment and Cell Motility protein 1 (ELMO1) of macrophages can also regulate intestinal inflammation induced by infectious agents through an autophagy pathway (Sarkar et al., 2017). Furthermore, autophagy can regulate the secretion of cytokines (including IL-1β, IL-1α, and IL-18) that are activated by the inflammasome. In turn, the sensing of pathogens by pattern-recognition receptors (PRRs) such as Toll-like receptors (TLRs) can promote autophagosome formation. Additionally, clearance of pathogens in the cytosol by autophagy may alleviate inflammasome activation. As post-translational modification event, ubiquitination was identified as a core process in pathogenesis and various host inflammatory responses. Ubiquitination is involved not only during autophagy, but also through the inflammatory signaling pathways and the mechanisms of antigen presentation in adaptive immune responses. *Salmonella* has also evolved some strategies to resist these host immune responses by regulating ubiquitination pathways.

**Figure 3:**
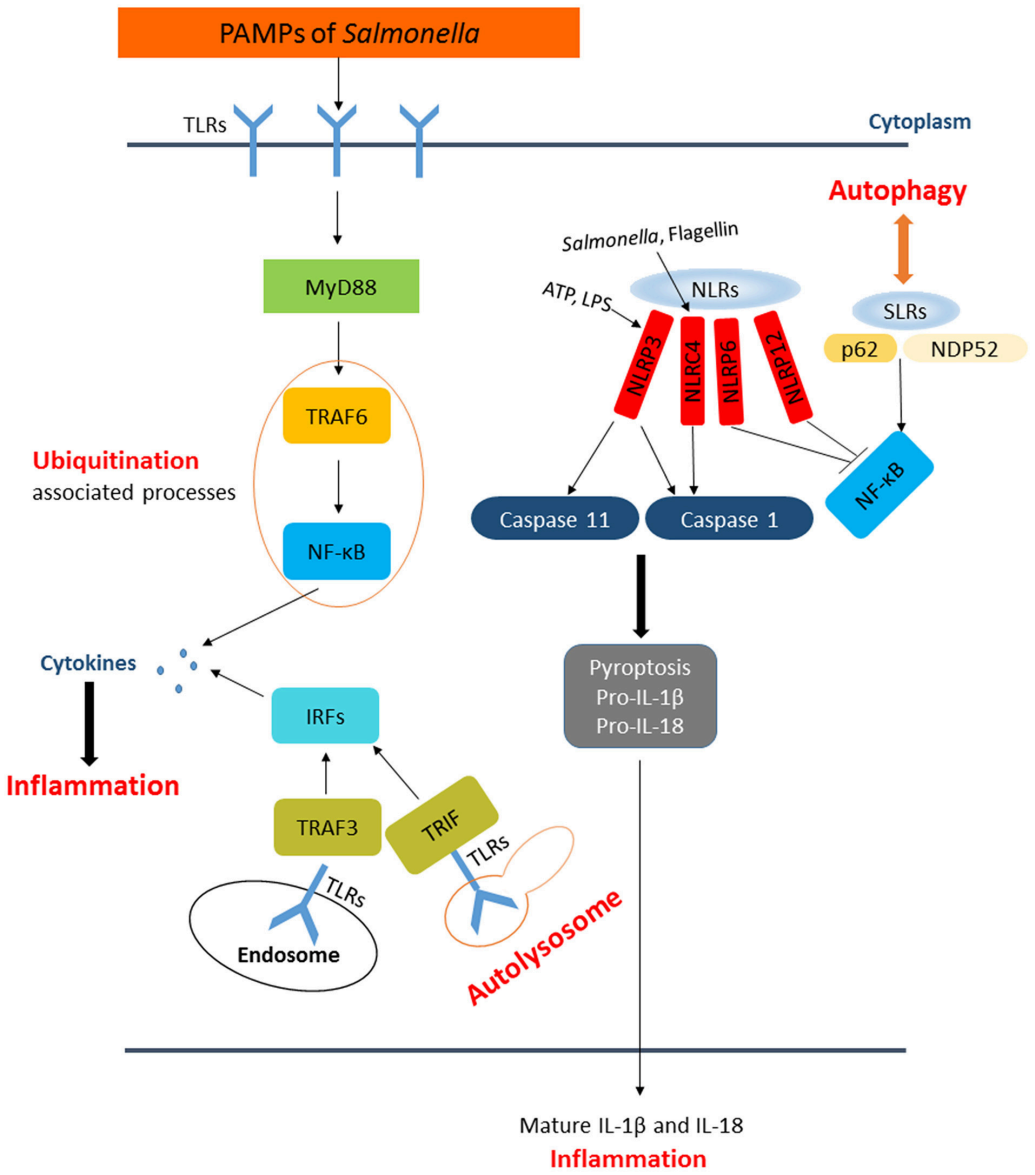
The paradigm of host inflammation responses to *Salmonella* and its interaction with autophagy as well as ubiquitination. PAMPs from *Salmonella* are recognized by membrane-bound TLRs in the extracellular space and within endosomes. The recognition of TLRs activate NF-κB and interferon-regulatory factors pathway to produce pro-inflammatory cytokines responding to *Salmonellae*. SLRs and NLRs are PRRs in the cytosol detecting intracellular pathogens. SLRs not only enable autophagy to degrade bacteria but also act as an inflammatory signaling platform. The activation of inflammasomes, which consist of caspase-1, NLRs, etc., can affect the inflammation in cytosol directly. NLRP3 and NLRC4 can lead to processing of caspase-1 or caspase-11, followed by secretion of pro-IL-1β and pro-IL-18 resulting in pyroptosis. NLRP6 and NLRP12 are also activated by *Salmonella* through unknown ligands that inhibiting NF-κB pathway.

There are numerous defense mechanisms in innate immune system to rapidly recognize and respond to pathogens, including the induction of inflammation which is a critical response to infections. The innate immune system detects pathogens and initiates inflammation through recognition of pathogen-associated molecular patterns (PAMPs) by PRRs, such as TLRs, C-type lectin receptors (CLRs), RIG-I like receptors (RLRs), Nod-like receptors (NLRs), and AIM2-like receptors (ALRs). While TLRs and CLRs are membrane-bound PRRs which detect bacteria in the extracellular space or within endosomes, the rest three receptors are PRRs resided in the cytosol detecting intracellular bacteria (Lamkanfi and Dixit, 2014). Sequestosome 1/p62-like receptors (SLRs) represent new family of innate immune receptors-a category of PRRs engaged in the recognition and capture of intracellular microbes (Figure 3) (Miao et al., 2010; Deretic, 2012). SLRs can initiate autophagy to eliminate intracellular microbes by direct capture and delivery of antimicrobial peptides, and serve as an inflammatory signaling platform. Multiple SLRs, such as p62 and NBR1, recognize either classical or branched Ub chains in association with or in vicinity of cytosolic *Salmonellae* (Thurston et al., 2009; Wild et al., 2011). A number of Ub recognized proteins, such as galectin 8, TBK1, and LC3, either independently or in combination, may lead to the recruitment of SLRs through their ubiquitin binding domains (UBDs) (Zhou and Zhu, 2015). NDP52 can recognize SLRs and also recruit the Ub tags, having dual functions on elimination of bacteria. It can not only recognize Ub coated *Salmonellae* but also recruit SLRs by the UBDs. Together with additional features of SLRs, it indicated that p62 can promote NF-κB induction and caspase-8 aggregation (Deretic, 2012).

NF-κB and interferon-regulatory factors (IRFs) pathways are two main signaling pathways activated for PRRs to promote pro-inflammatory and antimicrobial transcriptional responses, which play vital roles in inflammatory responses (Kopitar-Jerala, 2015). As a crucial pathway in response to *Salmonella* infection, there are several effectors regulating the inflammatory responses by modulating the NF-κB signaling pathway. In addition to the effectors we have mentioned above, the *Salmonella* virulence factor SrfA can also modulate inflammatory responses by increasing the activation of NF-κB signaling pathway (Lei et al., 2016). In addition, *S*. Typhimurium T3SS effector proteins PipA, GogA, and GtgA can specifically and redundantly target the components of the NF-κB signaling pathway leading to inflammation (Sun et al., 2016). The *Salmonella* T3SS-2 effector protein SpvD inhibits the NF-κB signaling pathway by inhibiting the nuclear transport of NF-kB p65 resulting in systemic growth of bacteria in mice (Rolhion et al., 2016). Remarkably, both NF-κB and IRFs pathways are greatly controlled by ubiquitination, and their activation can be affected by several proteins interfering with ubiquitination events (Zhou and Zhu, 2015). Besides its known role as an autophagy receptor, the protein OPTN was previously suggested as a control for the NF-κB and IRF-dependent inflammatory signaling. On one hand, being a homologous to NEMO (NF-κB essential modifier), OPTN competes with NEMO for binding IKKα and IKKβ to be ubiquitinated to inhibit TNF (tumor necrosis factor)-induced NF-κB signaling. On the other hand, the expression of OPTN is affected by NF-κB in response to TNF (Sudhakar et al., 2009). Interestingly, OPTN deficiency in mice reduced the secretion of pro-inflammatory cytokines such as TNF and IL-6, and diminished recruitment of neutrophils into the inflamed tissue (Chew et al., 2015).

Autophagy is one of the host innate immune responses against *Salmonella*. However, some research indicated that the induction of autophagy has a negative feedback process in inflammatory responses. Deretic et al. observed that the production of IL-1β and IL-18 is increased in the absence of functional ATG16L1 in a mouse model of Crohn's disease (Marchiando et al., 2013). It has been reported that the inhibition of autophagy was involved in pro-inflammatory cytokines production such as IL-1β and TNF-α in fish (Qin et al., 2016). Furthermore, macrophages infected with *Mycobacterium leprae* present high levels of autophagy activation but lower levels of pro-inflammatory cytokines (Ma et al., 2017). These studies suggested that autophagy induction could affect inflammatory responses, which will fuel advances in studies of *Salmonella* pathogenesis. The presence of specific inflammasome sensors in macrophages, such as NLRP3 and AIM2, could lead to the induction of autophagy. However, autophagosomes can selectively degrade specific inflammasome components, including AIM2, NLRP3, and ASC, etc. (Behnsen et al., 2015). The inflammasomes are intracellular complexes consisting of caspase-1, NLRs such as NLRP3 or NLRC4, and the adaptor molecule ASC (apoptosis-associated speck-like protein containing a CARD), which play a central role in innate immune defense against *S*. Typhimurium (Aachoui et al., 2013b; Man et al., 2014). Activation of inflammasomes are dependent on PAMPs recognition, ATP and intracellular LPS can activate the NLRP3 inflammasome, NLRC4 is activated by flagellin, dsDNA activates AIM2, and toxin-induced modifications of Rho GTPase is the activator of Pyrin inflammasome. Previous studies showed that NLRP3 and NLRC4 recruit ASC and caspase-1 in response to bacterial trigger, and both of NLRC4 and NLRP3 are activated during *Salmonella* infections (Man et al., 2014). Activation of these inflammasomes leads to caspase-1 activation and the secretion of the pro-inflammatory cytokines IL-1β and IL-18, resulting in a form of cell death known as pyroptosis (Figure 3) (Behnsen et al., 2015). Induction of flagellin expression leads to NLRC4-dependent pyroptosis during *S*. Typhimurium infection, which results in the exposure of released bacteria to infiltrating neutrophils (Lage et al., 2013). Man et al. showed that *Salmonella* infections activate a caspase-8–dependent pathway via NLRC4 that induces the formation of an ASC caspase-8–caspase-1 inflammasome complex (Man et al., 2014). A recent study proposed that NLRP3 is also responsible for sensing *Salmonellae* through a flagellin-independent mechanism, in which both NLRP3 and NLRC4 trigger ASC-dependent caspase-1 activation. However, the mechanism remains largely elusive (De Jong et al., 2014). Remarkably, the activation of inflammasome NLRP3 is restricted by NF-κB. While macrophages encounter NLRP3-inflammasome activators, mitochondria are damaged and following the release of inflammasome activating signals. On one hand, damaged mitochondria are rapidly ubiquitinated and subsequently eliminated by p62-dependent mitophagy. On the other hand, the mitophagy process restricts the activation of NLRP3. NF-κB playing a core role in this process, stimulating p62 gene transcription resulting in p62-dependent mitophagy and then restrains its own inflammation-promoting activity indirectly (Zhong et al., 2016). In contrast to NLRC4 and NLRP3, NLRP6 and NLRP12 negatively regulate the inflammatory responses during *Salmonella* infections. In addition, Anand et al. found that mice deficient in NLRP6 exhibit lower *Salmonellae* burden in organs, as well as an increase in the phosphorylation of IκB, indicating that this is related to the activation of NF-κB signaling (Anand et al., 2012; Zaki et al., 2014). However, the mechanisms by which *Salmonella* activates NLRP6 and NLRP12 remain to be determined.

The ubiquitination pathway and autophagy also have profound effects on innate immune responses through other mechanisms. The poly-Ub K63 chains have been involved in cell responses to danger signals through the TLRs, the interleukin-1 receptor (IL-1R) and the TNF (Ligeon et al., 2011). It was demonstrated that K63-linked Ub chains could mediate protein substrates targeting to the autophagy or ubiquitination pathway. The TNF receptor-associated factors (TRAF) family Ub ligases catalyze the formation of K63-linked Ub chains that activate NF-κB, ERK, JNK, and other signaling pathways (Yazlovitskaya et al., 2015). NOD2 (nucleotide oligomerization domain 2) and cytosolic pattern recognition receptors regulate the formation of K63-linked poly-Ub chains on the NEMO, thereby leading to activation of IKK and NF-κB pathway (Watanabe et al., 2014). TRIM38, an E3 Ub ligase, can regulate TLR3/4-mediated innate immunity and inflammatory responses. This protein catalyzed K48-linked poly-ubiquitination of the TLR3/4 adapter protein TIR domain which contains adapter-inducing IFN-β and promoted its proteasomal degradation in immune cells (Hu et al., 2015). As a pivotal NF-κB suppressor under TLRs, A20 can be captured by autophagy receptor p62 and then to be eliminated in the autophagosome, thus enhancing NF-κB activity (Kanayama et al., 2015). Moreover, Park et al. found that p62 is required for TNF and IL-1β production mediated by NOD2 (a cytosolic PRR) and up-regulates its signaling response through NF-κB activation. In contrast, p62 can induce MyD88 aggregation to suppress TLR signaling cascades (Park et al., 2013). LRRC25, a member of the LRR-containing protein family, revealed a new crosstalk between inflammation and autophagy. LRRC25 can promote the degradation of p65/RelA through autophagy, acting as an inhibitor of NF-κB signaling pathway (Feng et al., 2017).

**Figure 4:**
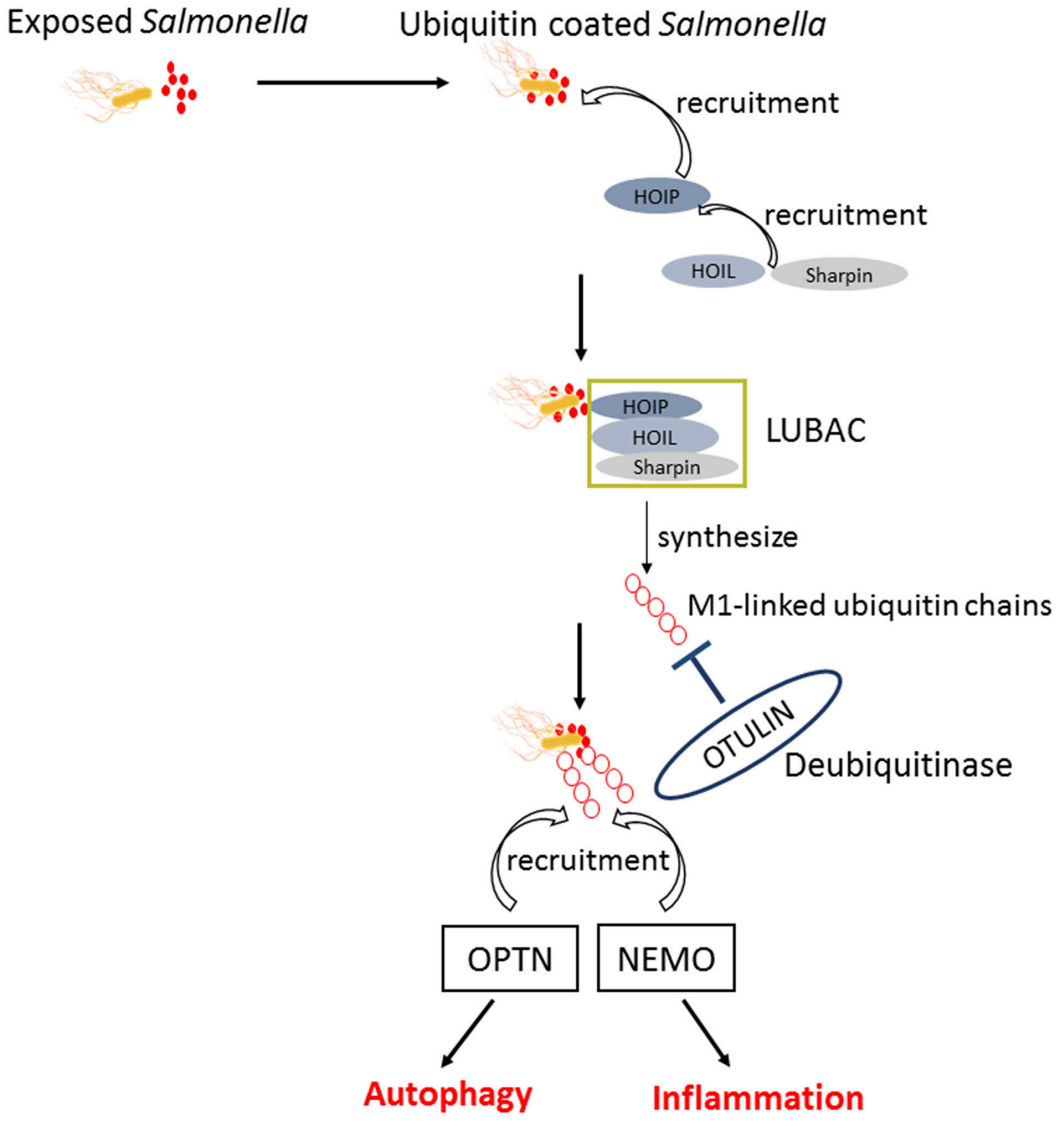
The similar activation process of autophagy and NF-κB pathway in response to *Salmonella*. Upon exposed in cytosol, *Salmonellae* were Ub coated and following the recruitment of HOIP, HOIL, and Sharpin. The combination of HOIP, HOIL, and Sharpin is termed as LUBAC, which can synthesize the M1-linked Ub chains. Ultimately, the M1-linked linear poly-Ub coated on *Salmonellae* recruit OPTN and NEMO to initiate autophagy and NF-κB signaling pathways, which play crucial roles in restrict proliferation of *Salmonellae* independently. OTULIN is a DUB which inhibits the recruitment of NEMO and ultimately NF-κB pathway by restraining the formation of M1-linked Ub chains.

Upon infection, *Salmonella* can not only trigger the autophagy flux in host cells but also evolve mechanisms for inhibiting this response. When T3SS-1 inflicted SCVs damage at the early stages of *S*. Typhimurium infection leading to bacterial exposure to the cytosol and initiating autophagy, autophagy promote repair of T3SS-1 inflicted damage to SCVs membrane conversely. Collectively, autophagy recognition of the damaged SCVs leads to bacterial elimination as well as SCVs repair. The balance between them relies on specific host responses and coordinate expression of bacterial virulent factors (Kreibich et al., 2015). *Salmonellae* recruit FAK (the host tyrosine kinase focal adhesion kinase) to the SCVs in a SPI-2 dependent way and then FAK suppresses autophagy, thereby preventing cell autonomous elimination and restrain the activation of innate TRIF-dependent type I interferon immune responses (Owen et al., 2016). The linear Ub chain assembly complex (LUBAC) was recently identified as a critical modulator of both innate immunity and inflammation signaling. LUBAC consists of SHANK-associated RH-domain–interacting protein (SHARPIN), heme-oxidized IRP2 Ub ligase-1 (HOIL-1), and HOIL-1–interacting protein (HOIP). Components of LUBAC were identified to control TLR3-mediated innate immunity (Zinngrebe et al., 2016). The M1 (methionine)-linked linear poly-Ub chain, synthesized by the E3 ligase LUBAC, serves as a novel signaling platform. Once coated with this platform, *S*. Typhimurium could be transmuted into antibacterial and pro-inflammatory status and recruit OPTN as well as NEMO to initiate autophagy process and NF-κB signaling pathway, respectively (Figure 4) (Noad et al., 2017). Additionally, E3 ligase ARIH1 can orchestrate the recognition of *Salmonella* and the activation of the host immune responses through two different mechanisms. It not only involves in Ub-coated bacteria, but also forms a network of ligases together with LRSAM1 and HOIP (Polajnar et al., 2017). Nevertheless, the DUB OTULIN is identified to restrain the formation of M1-linked Ub chains on the bacterial coat, then sequester the recruitment of NEMO and ultimately inhibit NF-κB pathway (Figure 4) (van Wijk et al., 2017). Intriguing but worrisome, E3 ligases involve in activation of host immune responses as well as inhibition of these processes by DUB, thus the interaction between Ub-related pathways and immune responses need to be further studied. A novel strategy of ubiquitination-dependent mechanism for *Salmonella* to manipulate host cells has also been revealed. *S*. Typhimurium T3SS effectors SopB and SopE2 can trigger ubiquitination of TRAF6, which is involved in STAT3 (Signal Transducer and Activator of Transcription 3) phosphorylation, a conductive signaling event in response to *S*. Typhimurium infection (Figure 3) (Ruan et al., 2017).

The inflammatory responses to *Salmonella* infection are a complex process, involving the common recognition of PAMPs by PRRs as well as autophagy and ubiquitination mechanisms. There are numerous strategies for the host to clear *Salmonellae*, while the bacteria also evolves mechanisms to subvert the host defenses. As reviewed in details of autophagy, ubiquitination and related inflammatory responses, a comprehensive understanding of host-*Salmonella* interaction was presented.

## Summary and future directions

*Salmonella* infections remain to be a threat to the public health owing to its transmissibility and pathogenicity. Despite copious amounts of advances in the understanding of autophagy and ubiquitination involved in responses to pathogens, how these processes are regulated to counteract *Salmonella* is incompletely defined. We therefore focus on autophagy and ubiquitination to clarify and summarize the host-pathogen interactions in detail. The related inflammatory responses have also been illuminated due to its closely association with the process of autophagy and ubiquitination pathways during bacterial infection. Since many bacteria have evolved strategies that allow for evasion of the autophagy pathway and host immune responses, it is important to identify the mechanism by which *Salmonella* inhibits autophagy and subverts the host responses. In the last ten years, more and more studies illuminate the interaction between *Salmonella* T3SS effector proteins and the host Ub pathways. Increasing evidence suggests that diverse mechanisms including molecular mimicry, formation of novel structures, and new enzymatic activities of effectors are involved in a crosstalk, leading with a modulation of autophagy and ubiquitination pathways. In this review, we summarized the connection between the Ub-proteasome system with autophagy and inflammatory responses in the context of *Salmonella* infection, in particular, the way effector proteins regulate the host defense by Ub and autophagy. The future challenge will be to address the commons of all the effectors, which may be helpful for eliminating *Salmonellae* in more efficient ways.

## Author contributions

RH and SW: outlined the manuscript; LW, JY, and HN: wrote the manuscript; LW, RH, and SW: edited the manuscript.

### Conflict of interest statement

The authors declare that the research was conducted in the absence of any commercial or financial relationships that could be construed as a potential conflict of interest.

## References

[B1] Aachoui Y. Leaf I. A. Hagar J. A. Fontana M. F. Campos C. G. Zak D. E.. (2013a). Caspase-11 protects against bacteria that escape the vacuole. Science 339, 975–978. 10.1126/science.1230751 23348507 PMC3697099

[B2] Aachoui Y. Sagulenko V. Miao E. A. Stacey K. J. (2013b). Inflammasome-mediated pyroptotic and apoptotic cell death, and defense against infection. Curr. Opin. Microbiol. 16, 319–326. 10.1016/j.mib.2013.04.004 23707339 PMC3742712

[B3] Anand P. K. Malireddi R. K. Lukens J. R. Vogel P. Bertin J. Lamkanfi M.. (2012). NLRP6 negatively regulates innate immunity and host defence against bacterial pathogens. Nature 488, 389–393. 10.1038/nature11250 22763455 PMC3422416

[B4] Ashida H. Mimuro H. Sasakawa C. (2015). Shigella manipulates host immune responses by delivering effector proteins with specific roles. Front. Immunol. 6:219. 10.3389/fimmu.2015.00219 25999954 PMC4423471

[B5] Bayer-Santos E. Durkin C. H. Rigano L. A. Kupz A. Alix E. Cerny O.. (2016). The *Salmonella* effector SteD mediates MARCH8-Dependent ubiquitination of MHC II molecules and inhibits T cell activation. Cell Host Microbe 20, 584–595. 10.1016/j.chom.2016.10.007 27832589 PMC5104694

[B6] Behnsen J. Perez-Lopez A. Nuccio S. P. Raffatellu M. (2015). Exploiting host immunity: the *Salmonella* paradigm. Trends Immunol. 36, 112–120. 10.1016/j.it.2014.12.003 25582038 PMC4323876

[B7] Beuzón C. R. Meresse S. Unsworth K. E. Ruiz-Albert J. Garvis S. Waterman S. R.. (2000). *Salmonella* maintains the integrity of its intracellular vacuole through the action of SifA. EMBO J. 19, 3235–3249. 10.1093/emboj/19.13.3235 10880437 PMC313946

[B8] Bhattacharya A. Wei Q. Shin J. N. Abdel Fattah E. Bonilla D. L. Xiang Q.. (2015). Autophagy is required for Neutrophil-Mediated inflammation. Cell Rep. 12, 1731–1739. 10.1016/j.celrep.2015.08.019 26344765

[B9] Bhavsar A. P. Brown N. F. Stoepel J. Wiermer M. Martin D. D. Hsu K. J.. (2013). The *Salmonella* type III effector SspH2 specifically exploits the NLR co-chaperone activity of SGT1 to subvert immunity. PLoS Pathog. 9:e1003518. 10.1371/journal.ppat.1003518 23935490 PMC3723637

[B10] Birmingham C. L. Brumell J. H. (2006). Autophagy recognizes intracellular *Salmonella* enterica serovar Typhimurium in damaged vacuoles. Autophagy 2, 156–158. 10.4161/auto.2825 16874057

[B11] Burkinshaw B. J. Strynadka N. C. (2014). Assembly and structure of the T3SS. Biochim. Biophys. Acta 1843, 1649–1663. 10.1016/j.bbamcr.2014.01.035 24512838

[B12] Cemma M. Kim P. K. Brumell J. H. (2011). The ubiquitin-binding adaptor proteins p62/SQSTM1 and NDP52 are recruited independently to bacteria-associated microdomains to target *Salmonella* to the autophagy pathway. Autophagy 7, 341–345. 10.4161/auto.7.3.14046 21079414 PMC3060414

[B13] Cha-Molstad H. Yu J. E. Feng Z. Lee S. H. Kim J. G. Yang P.. (2017). p62/SQSTM1/Sequestosome-1 is an N-recognin of the N-end rule pathway which modulates autophagosome biogenesis. Nat. Commun. 8:102. 10.1038/s41467-017-00085-7 28740232 PMC5524641

[B14] Chew T. S. O'Shea N. R. Sewell G. W. Oehlers S. H. Mulvey C. M. Crosier P. S.. (2015). Optineurin deficiency in mice contributes to impaired cytokine secretion and neutrophil recruitment in bacteria-driven colitis. Dis. Model. Mech. 8, 817–829. 10.1242/dmm.020362 26044960 PMC4527293

[B15] Coombes B. K. Lowden M. J. Bishop J. L. Wickham M. E. Brown N. F. Duong N.. (2007). SseL is a salmonella-specific translocated effector integrated into the SsrB-controlled *Salmonella* pathogenicity island 2 type III secretion system. Infect. Immun. 75, 574–580. 10.1128/IAI.00985-06 17158898 PMC1828504

[B16] Cordero-Alba M. Ramos-Morales F. (2014). Patterns of expression and translocation of the ubiquitin ligase SlrP in *Salmonella* enterica serovar Typhimurium. J. Bacteriol. 196, 3912–3922. 10.1128/JB.02158-14 25182488 PMC4248824

[B17] De Jong H. K. Koh G. C. van Lieshout M. H. Roelofs J. J. van Dissel J. T. van der Poll T.. (2014). Limited role for ASC and NLRP3 during *in vivo Salmonella typhimurium* infection. BMC Immunol. 15:30. 10.1186/s12865-014-0030-7 25115174 PMC4243774

[B18] Deretic V. (2011). Autophagy in immunity and cell-autonomous defense against intracellular microbes. Immunol. Rev. 240, 92–104. 10.1111/j.1600-065X.2010.00995.x 21349088 PMC3057454

[B19] Deretic V. (2012). Autophagy as an innate immunity paradigm: expanding the scope and repertoire of pattern recognition receptors. Curr. Opin. Immunol. 24, 21–31. 10.1016/j.coi.2011.10.006 22118953 PMC3288884

[B20] Diao J. Zhang Y. Huibregtse J. M. Zhou D. Chen J. (2008). Crystal structure of SopA, a *Salmonella* effector protein mimicking a eukaryotic ubiquitin ligase. Nat. Struct. Mol. Biol. 15, 65–70. 10.1038/nsmb1346 18066077

[B21] Eldridge M. J. Sanchez-Garrido J. Hoben G. F. Goddard P. J. Shenoy A. R. (2017). The Atypical ubiquitin E2 conjugase UBE2L3 is an indirect Caspase-1 target and controls IL-1beta secretion by inflammasomes. Cell Rep. 18, 1285–1297. 10.1016/j.celrep.2017.01.015 28147281 PMC5300903

[B22] Feng Y. Duan T. Du Y. Jin S. Wang M. Cui J.. (2017). LRRC25 functions as an inhibitor of NF-kappaB signaling pathway by promoting p65/RelA for autophagic degradation. Sci. Rep. 7:13448. 10.1038/s41598-017-12573-3 29044191 PMC5647368

[B23] Fiskin E. Bhogaraju S. Herhaus L. Kalayil S. Hahn M. Dikic I. (2017). Structural basis for the recognition and degradation of host TRIM proteins by *Salmonella* effector SopA. Nat. Commun. 8:14004. 10.1038/ncomms14004 28084320 PMC5241803

[B24] Fiskin E. Bionda T. Dikic I. Behrends C. (2016). Global analysis of host and bacterial ubiquitinome in response to *Salmonella typhimurium* infection. Mol. Cell 62, 967–981. 10.1016/j.molcel.2016.04.015 27211868

[B25] Franco L. H. Nair V. R. Scharn C. R. Xavier R. J. Torrealba J. R. Shiloh M. U.. (2017). The ubiquitin ligase Smurf1 functions in selective autophagy of *Mycobacterium tuberculosis* and Anti-tuberculous host defense. Cell Host Microbe 21, 59–72. 10.1016/j.chom.2016.11.002 28017659 PMC5699477

[B26] Fujita N. Yoshimori T. (2011). Ubiquitination-mediated autophagy against invading bacteria. Curr. Opin. Cell Biol. 23, 492–497. 10.1016/j.ceb.2011.03.003 21450448

[B27] Galbas T. Raymond M. Sabourin A. Bourgeois-Daigneault M. C. Guimont-Desrochers F. Yun T. J.. (2017). MARCH1 E3 ubiquitin ligase dampens the innate inflammatory response by modulating monocyte functions in mice. J. Immunol. 198, 852–861. 10.4049/jimmunol.1601168 27940660

[B28] Giacomodonato M. N. Noto Llana M. Aya Castaneda Mdel R. Buzzola F. R. Sarnacki S. H. Cerquetti M. C. (2014). AvrA effector protein of *Salmonella* enterica serovar Enteritidis is expressed and translocated in mesenteric lymph nodes at late stages of infection in mice. Microbiology 160, 1191–1199. 10.1099/mic.0.077115-0 24705228 PMC4811645

[B29] Gomes L. C. Dikic I. (2014). Autophagy in antimicrobial immunity. Mol. Cell 54, 224–233. 10.1016/j.molcel.2014.03.009 24766886

[B30] Grumati P. Dikic I. (2017). Ubiquitin signaling and autophagy. J. Biol. Chem. [Epub ahead of print]. 10.1074/jbc.TM117.000117 29187595 PMC5900779

[B31] Heath R. J. Goel G. Baxt L. A. Rush J. S. Mohanan V. Paulus G. L.. (2016). RNF166 Determines recruitment of adaptor proteins during antibacterial autophagy. Cell Rep. 17, 2183–2194. 10.1016/j.celrep.2016.11.005 27880896 PMC5192565

[B32] Heo J. M. Ordureau A. Paulo J. A. Rinehart J. Harper J. W. (2015). The PINK1-PARKIN mitochondrial ubiquitylation pathway drives a program of OPTN/NDP52 recruitment and TBK1 activation to promote mitophagy. Mol. Cell 60, 7–20. 10.1016/j.molcel.2015.08.016 26365381 PMC4592482

[B33] Hu M. M. Xie X. Q. Yang Q. Liao C. Y. Ye W. Lin H.. (2015). TRIM38 negatively regulates TLR3/4-Mediated innate immune and inflammatory responses by Two sequential and distinct mechanisms. J. Immunol. 195, 4415–4425. 10.4049/jimmunol.1500859 26392463

[B34] Ishimura R. Tanaka K. Komatsu M. (2014). Dissection of the role of p62/Sqstm1 in activation of Nrf2 during xenophagy. FEBS Lett. 588, 822–828. 10.1016/j.febslet.2014.01.045 24492006

[B35] Ivanov S. Roy C. R. (2009). NDP52: the missing link between ubiquitinated bacteria and autophagy. Nat. Immunol. 10, 1137–1139. 10.1038/ni1109-1137 19841643 PMC2803095

[B36] Kamanova J. Sun H. Lara-Tejero M. Galan J. E. (2016). The *Salmonella* effector protein sopa modulates innate immune responses by targeting TRIM E3 ligase family members. PLoS Pathog. 12:e1005552. 10.1371/journal.ppat.1005552 27058235 PMC4825927

[B37] Kanayama M. Inoue M. Danzaki K. Hammer G. He Y. W. Shinohara M. L. (2015). Autophagy enhances NFkappaB activity in specific tissue macrophages by sequestering A20 to boost antifungal immunity. Nat. Commun. 6:5779. 10.1038/ncomms6779 25609235 PMC4304414

[B38] Keestra-Gounder A. M. Tsolis R. M. Baumler A. J. (2015). Now you see me, now you don't: the interaction of *Salmonella* with innate immune receptors. Nat. Rev. Microbiol. 13, 206–216. 10.1038/nrmicro3428 25749454

[B39] Keszei A. F. Tang X. McCormick C. Zeqiraj E. Rohde J. R. Tyers M.. (2014). Structure of an SspH1-PKN1 complex reveals the basis for host substrate recognition and mechanism of activation for a bacterial E3 ubiquitin ligase. Mol. Cell. Biol. 34, 362–373. 10.1128/MCB.01360-13 24248594 PMC3911519

[B40] Kopitar-Jerala N. (2015). Innate immune response in brain, NF-Kappa B signaling and cystatins. Front. Mol. Neurosci. 8:73. 10.3389/fnmol.2015.00073 26696821 PMC4673337

[B41] Kreibich S. Emmenlauer M. Fredlund J. Ramo P. Munz C. Dehio C.. (2015). Autophagy proteins promote repair of endosomal membranes damaged by the *Salmonella* type Three secretion system 1. Cell Host Microbe 18, 527–537. 10.1016/j.chom.2015.10.015 26567507

[B42] Kummari E. Alugubelly N. Hsu C. Y. Dong B. Nanduri B. Edelmann M. J. (2015). Activity-Based proteomic profiling of deubiquitinating enzymes in *Salmonella*-Infected macrophages leads to identification of putative function of UCH-L5 in inflammasome regulation. PLoS ONE 10:e0135531 10.1371/journal.pone.0138635 26267804 PMC4534353

[B43] Lage S. L. Buzzo C. L. Amaral E. P. Matteucci K. C. Massis L. M. Icimoto M. Y.. (2013). Cytosolic flagellin-induced lysosomal pathway regulates inflammasome-dependent and -independent macrophage responses. Proc. Natl. Acad. Sci. U.S.A. 110, E3321– E3330. 10.1073/pnas.1305316110 23942123 PMC3761566

[B44] Lamkanfi M. Dixit V. M. (2014). Mechanisms and functions of inflammasomes. Cell 157, 1013–1022. 10.1016/j.cell.2014.04.007 24855941

[B45] Lapaque N. Hutchinson J. L. Jones D. C. Meresse S. Holden D. W. Trowsdale J.. (2009). *Salmonella* regulates polyubiquitination and surface expression of MHC class II antigens. Proc. Natl. Acad. Sci. U.S.A. 106, 14052–14057. 10.1073/pnas.0906735106 19666567 PMC2721820

[B46] Lee Y. Chou T. F. Pittman S. K. Keith A. L. Razani B. Weihl C. C. (2017). Keap1/Cullin3 modulates p62/SQSTM1 activity via UBA domain ubiquitination. Cell Rep. 19, 188–202. 10.1016/j.celrep.2017.03.030 28380357 PMC5395095

[B47] Lei L. Wang W. Xia C. Liu F. (2016). *Salmonella* virulence factor SsrAB regulated factor modulates inflammatory responses by enhancing the activation of NF-kappaB signaling pathway. J. Immunol. 196, 792–802. 10.4049/jimmunol.1500679 26673132

[B48] Le Negrate G. Faustin B. Welsh K. Loeffler M. Krajewska M. Hasegawa P.. (2008). *Salmonella* secreted factor L deubiquitinase of *Salmonella typhimurium* inhibits NF-kappaB, suppresses IkappaBalpha ubiquitination and modulates innate immune responses. J. Immunol. 180, 5045–5056. 10.4049/jimmunol.180.7.5045 18354230

[B49] Ligeon L. A. Temime-Smaali N. Lafont F. (2011). Ubiquitylation and autophagy in the control of bacterial infections and related inflammatory responses. Cell. Microbiol. 13, 1303–1311. 10.1111/j.1462-5822.2011.01628.x 21740497

[B50] Lopez-Castejon G. Luheshi N. M. Compan V. High S. Whitehead R. C. Flitsch S.. (2013). Deubiquitinases regulate the activity of caspase-1 and interleukin-1beta secretion via assembly of the inflammasome. J. Biol. Chem. 288, 2721–2733. 10.1074/jbc.M112.422238 23209292 PMC3554938

[B51] Ma Y. Zhang L. Lu J. Shui T. Chen J. Yang J.. (2017). A negative feedback loop between autophagy and immune responses in *Mycobacterium leprae* infection. DNA Cell Biol. 36, 1–9. 10.1089/dna.2016.3446 27854511

[B52] Man S. M. Hopkins L. J. Nugent E. Cox S. Gluck I. M. Tourlomousis P.. (2014). Inflammasome activation causes dual recruitment of NLRC4 and NLRP3 to the same macromolecular complex. Proc. Natl. Acad. Sci. U.S.A. 111, 7403–7408. 10.1073/pnas.1402911111 24803432 PMC4034195

[B53] Marchiando A. M. Ramanan D. Ding Y. Gomez L. E. Hubbard-Lucey V. M. Maurer K.. (2013). A deficiency in the autophagy gene Atg16L1 enhances resistance to enteric bacterial infection. Cell Host Microbe 14, 216–224. 10.1016/j.chom.2013.07.013 23954160 PMC3825684

[B54] Martinon F. Tschopp J. (2007). Inflammatory caspases and inflammasomes: master switches of inflammation. Cell Death Differ. 14, 10–22. 10.1038/sj.cdd.4402038 16977329

[B55] Mesquita F. S. Thomas M. Sachse M. Santos A. J. Figueira R. Holden D. W. (2012). The *Salmonella* deubiquitinase SseL inhibits selective autophagy of cytosolic aggregates. PLoS Pathog. 8:e1002743. 10.1371/journal.ppat.1002743 22719249 PMC3375275

[B56] Miao E. A. Leaf I. A. Treuting P. M. Mao D. P. Dors M. Sarkar A.. (2010). Caspase-1-induced pyroptosis is an innate immune effector mechanism against intracellular bacteria. Nat. Immunol. 11, 1136–1142. 10.1038/ni.1960 21057511 PMC3058225

[B57] Noad J. von der Malsburg A. Pathe C. Michel M. A. Komander D. Randow F. (2017). LUBAC-synthesized linear ubiquitin chains restrict cytosol-invading bacteria by activating autophagy and NF-kappaB. Nat. Microbiol. 2:17063. 10.1038/nmicrobiol.2017.63 28481331 PMC5576533

[B58] Ohlson M. B. Huang Z. Alto N. M. Blanc M. P. Dixon J. E. Chai J.. (2008). Structure and function of *Salmonella* SifA indicate that its interactions with SKIP, SseJ, and RhoA family GTPases induce endosomal tubulation. Cell Host Microbe 4, 434–446. 10.1016/j.chom.2008.08.012 18996344 PMC2658612

[B59] Owen K. A. Anderson C. J. Casanova J. E. (2016). *Salmonella* suppresses the TRIF-Dependent Type I interferon response in macrophages. MBio 7, e02051–e02015. 10.1128/mBio.02051-15 26884434 PMC4791850

[B60] Owen K. A. Casanova J. E. (2015). *Salmonella* manipulates autophagy to “Serve and Protect”. Cell Host Microbe 18, 517–519. 10.1016/j.chom.2015.10.020 26567504

[B61] Park S. Ha S. D. Coleman M. Meshkibaf S. Kim S. O. (2013). p62/SQSTM1 enhances NOD2-mediated signaling and cytokine production through stabilizing NOD2 oligomerization. PLoS ONE 8:e57138. 10.1371/journal.pone.0057138 23437331 PMC3577775

[B62] Patel J. C. Hueffer K. Lam T. T. Galan J. E. (2009). Diversification of a *Salmonella* virulence protein function by ubiquitin-dependent differential localization. Cell 137, 283–294. 10.1016/j.cell.2009.01.056 19379694 PMC2673707

[B63] Peng H. Yang J. Li G. You Q. Han W. Li T.. (2017). Ubiquitylation of p62/sequestosome1 activates its autophagy receptor function and controls selective autophagy upon ubiquitin stress. Cell Res. 27, 657–674. 10.1038/cr.2017.40 28322253 PMC5520855

[B64] Pilar A. V. Reid-Yu S. A. Cooper C. A. Mulder D. T. Coombes B. K. (2012). GogB is an anti-inflammatory effector that limits tissue damage during *Salmonella* infection through interaction with human FBXO22 and Skp1. PLoS Pathog. 8:e1002773. 10.1371/journal.ppat.1002773 22761574 PMC3386239

[B65] Polajnar M. Dietz M. S. Heilemann M. Behrends C. (2017). Expanding the host cell ubiquitylation machinery targeting cytosolic *Salmonella*. EMBO Rep. 18, 1572–1585. 10.15252/embr.201643851 28784601 PMC5579355

[B66] Qin L. Wang X. Zhang S. Feng S. Yin L. Zhou H. (2016). Lipopolysaccharide-induced autophagy participates in the control of pro-inflammatory cytokine release in grass carp head kidney leukocytes. Fish Shellfish Immunol. 59, 389–397. 10.1016/j.fsi.2016.11.010 27826112

[B67] Quezada C. M. Hicks S. W. Galan J. E. Stebbins C. E. (2009). A family of *Salmonella* virulence factors functions as a distinct class of autoregulated E3 ubiquitin ligases. Proc. Natl. Acad. Sci. U.S.A. 106, 4864–4869. 10.1073/pnas.0811058106 19273841 PMC2653562

[B68] Radtke A. L. Delbridge L. M. Balachandran S. Barber G. N. O'Riordan M. X. (2007). TBK1 protects vacuolar integrity during intracellular bacterial infection. PLoS Pathog. 3:e29. 10.1371/journal.ppat.0030029 17335348 PMC1808071

[B69] Rai A. Pathak D. Thakur S. Singh S. Dubey A. K. Mallik R. (2016). Dynein clusters into lipid microdomains on phagosomes to drive rapid transport toward lysosomes. Cell 164, 722–734. 10.1016/j.cell.2015.12.054 26853472 PMC4752818

[B70] Ria R. Todoerti K. Berardi S. Coluccia A. M. De Luisi A. Mattioli M.. (2009). Gene expression profiling of bone marrow endothelial cells in patients with multiple myeloma. Clin. Cancer Res. 15, 5369–5378. 10.1158/1078-0432.CCR-09-0040 19690192

[B71] Riffelmacher T. Richter F. C. Simon A. K. (2017). Autophagy dictates metabolism and differentiation of inflammatory immune cells. Autophagy 14, 1–8. 10.1080/15548627.2017.1362525 28806133 PMC5902226

[B72] Rogov V. V. Suzuki H. Fiskin E. Wild P. Kniss A. Rozenknop A.. (2013). Structural basis for phosphorylation-triggered autophagic clearance of *Salmonella*. Biochem. J. 454, 459–466. 10.1042/BJ20121907 23805866

[B73] Rohde J. R. Breitkreutz A. Chenal A. Sansonetti P. J. Parsot C. (2007). Type III secretion effectors of the IpaH family are E3 ubiquitin ligases. Cell Host Microbe 1, 77–83. 10.1016/j.chom.2007.02.002 18005683

[B74] Rolhion N. Furniss R. C. Grabe G. Ryan A. Liu M. Matthews S. A.. (2016). Inhibition of nuclear transport of NF-kB p65 by the *Salmonella* Type III secretion system effector SpvD. PLoS Pathog. 12:e1005653. 10.1371/journal.ppat.1005653 27232334 PMC4883751

[B75] Rossignol A. Roche S. M. Virlogeux-Payant I. Wiedemann A. Grepinet O. Fredlund J.. (2014). Deciphering why *Salmonella* Gallinarum is less invasive *in vitro* than *Salmonella* Enteritidis. Vet. Res. 45:81. 10.1186/s13567-014-0081-z 25175996 PMC4154518

[B76] Ruan H. H. Li Y. Zhang X. X. Liu Q. Ren H. Zhang K. S.. (2014). Identification of TRAF6 as a ubiquitin ligase engaged in the ubiquitination of SopB, a virulence effector protein secreted by *Salmonella typhimurium*. Biochem. Biophys. Res. Commun. 447, 172–177. 10.1016/j.bbrc.2014.03.126 24704445

[B77] Ruan H. H. Zhang Z. Wang S. Y. Nickels L. M. Tian L. Qiao J. J.. (2017). Tumor necrosis factor receptor-associated factor 6 (TRAF6) mediates ubiquitination-dependent STAT3 activation upon *Salmonella typhimurium* infection. Infect. Immun. 85:e00081–17. 10.1128/IAI.00081-17 28507064 PMC5520427

[B78] Rytkönen A. Poh J. Garmendia J. Boyle C. Thompson A. Liu M.. (2007). SseL, a *Salmonella* deubiquitinase required for macrophage killing and virulence. Proc. Natl. Acad. Sci. U.S.A. 104, 3502–3507. 10.1073/pnas.0610095104 17360673 PMC1802004

[B79] Salomon D. Orth K. (2013). What pathogens have taught us about posttranslational modifications. Cell Host Microbe 14, 269–279. 10.1016/j.chom.2013.07.008 24034613 PMC5785091

[B80] Sarkar A. Tindle C. Pranadinata R. F. Reed S. Eckmann L. Stappenbeck T. S.. (2017). ELMO1 regulates the induction of autophagy and bacterial clearance during enteric infection. J. Infect. Dis. 216, 1655–1666. 10.1093/infdis/jix528 29029244 PMC5853658

[B81] Scanu T. Spaapen R. M. Bakker J. M. Pratap C. B. Wu L. E. Hofland I.. (2015). *Salmonella* manipulation of host signaling pathways provokes cellular transformation associated with Gallbladder Carcinoma. Cell Host Microbe 17, 763–774. 10.1016/j.chom.2015.05.002 26028364

[B82] Singh V. Finke-Isami J. Hopper-Chidlaw A. C. Schwerk P. Thompson A. Tedin K. (2017). *Salmonella* Co-opts host cell Chaperone-mediated autophagy for intracellular growth. J. Biol. Chem. 292, 1847–1864. 10.1074/jbc.M116.759456 27932462 PMC5290957

[B83] Song M. Sukovich D. J. Ciccarelli L. Mayr J. Fernandez-Rodriguez J. Mirsky E. A.. (2017). Control of type III protein secretion using a minimal genetic system. Nat. Commun. 8:14737. 10.1038/ncomms14737 28485369 PMC5436071

[B84] Sotthibundhu A. McDonagh K. von Kriegsheim A. Garcia-Munoz A. Klawiter A. Thompson K.. (2016). Rapamycin regulates autophagy and cell adhesion in induced pluripotent stem cells. Stem Cell Res. Ther. 7:166. 10.1186/s13287-016-0425-x 27846905 PMC5109678

[B85] Sudhakar C. Nagabhushana A. Jain N. Swarup G. (2009). NF-kappaB mediates tumor necrosis factor alpha-induced expression of optineurin, a negative regulator of NF-kappaB. PLoS ONE 4:e5114. 10.1371/journal.pone.0005114 19340308 PMC2660438

[B86] Sun A. Wei J. Childress C. Shaw J. H. Peng K. Shao G.. (2017). The E3 ubiquitin ligase NEDD4 is an LC3-interactive protein and regulates autophagy. Autophagy 13, 522–537. 10.1080/15548627.2016.1268301 28085563 PMC5361608

[B87] Sun H. Kamanova J. Lara-Tejero M. Galan J. E. (2016). A family of *Salmonella* Type III secretion effector proteins selectively targets the NF-kappaB signaling pathway to preserve host homeostasis. PLoS Pathog. 12:e1005484. 10.1371/journal.ppat.1005484 26933955 PMC4775039

[B88] Szeto J. Kaniuk N. A. Canadien V. Nisman R. Mizushima N. Yoshimori T.. (2006). ALIS are stress-induced protein storage compartments for substrates of the proteasome and autophagy. Autophagy 2, 189–199. 10.4161/auto.2731 16874109

[B89] Teo W. X. Kerr M. C. Teasdale R. D. (2016). MTMR4 is required for the stability of the *Salmonella*-Containing vacuole. Front. Cell. Infect. Microbiol. 6:91. 10.3389/fcimb.2016.00091 27625994 PMC5003867

[B90] Thurston T. L. Boyle K. B. Allen M. Ravenhill B. J. Karpiyevich M. Bloor S.. (2016). Recruitment of TBK1 to cytosol-invading *Salmonella* induces WIPI2-dependent antibacterial autophagy. EMBO J. 35, 1779–1792. 10.15252/embj.201694491 27370208 PMC5010046

[B91] Thurston T. L. Ryzhakov G. Bloor S. von Muhlinen N. Randow F. (2009). The TBK1 adaptor and autophagy receptor NDP52 restricts the proliferation of ubiquitin-coated bacteria. Nat. Immunol. 10, 1215–1221. 10.1038/ni.1800 19820708

[B92] Thurston T. L. Wandel M. P. von Muhlinen N. Foeglein A. Randow F. (2012). Galectin 8 targets damaged vesicles for autophagy to defend cells against bacterial invasion. Nature 482, 414–418. 10.1038/nature10744 22246324 PMC3343631

[B93] Tumbarello D. A. Manna P. T. Allen M. Bycroft M. Arden S. D. Kendrick-Jones J.. (2015). The autophagy receptor TAX1BP1 and the molecular motor myosin VI are required for clearance of *Salmonella typhimurium* by autophagy. PLoS Pathog. 11:e1005174. 10.1371/journal.ppat.1005174 26451915 PMC4599966

[B94] van Wijk S. J. L. Fricke F. Herhaus L. Gupta J. Hotte K. Pampaloni F.. (2017). Linear ubiquitination of cytosolic *Salmonella typhimurium* activates NF-kappaB and restricts bacterial proliferation. Nat. Microbiol. 2:17066. 10.1038/nmicrobiol.2017.66 28481361

[B95] Verlhac P. Gregoire I. P. Azocar O. Petkova D. S. Baguet J. Viret C.. (2015). Autophagy receptor NDP52 regulates pathogen-containing autophagosome maturation. Cell Host Microbe 17, 515–525. 10.1016/j.chom.2015.02.008 25771791

[B96] Walczak H. Iwai K. Dikic I. (2012). Generation and physiological roles of linear ubiquitin chains. BMC Biol. 10:23. 10.1186/1741-7007-10-23 22420778 PMC3305636

[B97] Watanabe T. Asano N. Meng G. Yamashita K. Arai Y. Sakurai T.. (2014). NOD2 downregulates colonic inflammation by IRF4-mediated inhibition of K63-linked polyubiquitination of RICK and TRAF6. Mucosal Immunol. 7, 1312–1325. 10.1038/mi.2014.19 24670424 PMC4177019

[B98] Weidberg H. Elazar Z. (2011). TBK1 mediates crosstalk between the innate immune response and autophagy. Sci. Signal. 4:pe39. 10.1126/scisignal.2002355 21868362

[B99] Wild P. Farhan H. McEwan D. G. Wagner S. Rogov V. V. Brady N. R.. (2011). Phosphorylation of the autophagy receptor optineurin restricts *Salmonella* growth. Science 333, 228–233. 10.1126/science.1205405 21617041 PMC3714538

[B100] Yang Z. Soderholm A. Lung T. W. Giogha C. Hill M. M. Brown N. F.. (2015). SseK3 Is a *Salmonella* effector that binds TRIM32 and modulates the Host's NF-kappaB signalling activity. PLoS ONE 10:e0138529. 10.1371/journal.pone.0138529 26394407 PMC4579058

[B101] Yazlovitskaya E. M. Tseng H. Y. Viquez O. Tu T. Mernaugh G. McKee K. K.. (2015). Integrin alpha3beta1 regulates kidney collecting duct development via TRAF6-dependent K63-linked polyubiquitination of Akt. Mol. Biol. Cell 26, 1857–1874. 10.1091/mbc.E14-07-1203 25808491 PMC4436831

[B102] Zaki M. H. Man S. M. Vogel P. Lamkanfi M. Kanneganti T. D. (2014). *Salmonella* exploits NLRP12-dependent innate immune signaling to suppress host defenses during infection. Proc. Natl. Acad. Sci. U.S.A. 111, 385–390. 10.1073/pnas.1317643111 24347638 PMC3890849

[B103] Zhang Y. Higashide W. Dai S. Sherman D. M. Zhou D. (2005). Recognition and ubiquitination of *Salmonella* type III effector SopA by a ubiquitin E3 ligase, HsRMA1. J. Biol. Chem. 280, 38682–38688. 10.1074/jbc.M506309200 16176924

[B104] Zhang Y. Higashide W. M. McCormick B. A. Chen J. Zhou D. (2006). The inflammation-associated *Salmonella* SopA is a HECT-like E3 ubiquitin ligase. Mol. Microbiol. 62, 786–793. 10.1111/j.1365-2958.2006.05407.x 17076670

[B105] Zhao W. Moest T. Zhao Y. Guilhon A. A. Buffat C. Gorvel J. P.. (2015). The *Salmonella* effector protein SifA plays a dual role in virulence. Sci. Rep. 5:12979. 10.1038/srep12979 26268777 PMC4534788

[B106] Zhong Z. Umemura A. Sanchez-Lopez E. Liang S. Shalapour S. Wong J.. (2016). NF-kappaB restricts inflammasome activation via elimination of damaged mitochondria. Cell 164, 896–910. 10.1016/j.cell.2015.12.057 26919428 PMC4769378

[B107] Zhou Y. Zhu Y. (2015). Diversity of bacterial manipulation of the host ubiquitin pathways. Cell. Microbiol. 17, 26–34. 10.1111/cmi.12384 25339545

[B108] Zinngrebe J. Rieser E. Taraborrelli L. Peltzer N. Hartwig T. Ren H.. (2016). LUBAC deficiency perturbs TLR3 signaling to cause immunodeficiency and autoinflammation. J. Exp. Med. 213, 2671–2689. 10.1084/jem.20160041 27810922 PMC5110014

